# Forecasting Neonatal Mortality in Ethiopia to Assess Progress Toward National and International Reduction Targets Using Classical Techniques and Deep Learning: Time-Series Forecasting Study

**DOI:** 10.2196/66798

**Published:** 2025-08-25

**Authors:** Shimels Derso Kebede

**Affiliations:** 1 Department of Health Informatics, School of Public Health, College of Medicine and Health Science, Wollo University, South Wollo Zone, Amhara Region, Dessie, Ethiopia, 251 940219818

**Keywords:** neonatal mortality, time series, forecasting, deep learning, machine learning, health sector transformation plan, deep learning model, Ethiopia

## Abstract

**Background:**

Neonatal disease and its outcomes are important indicators for a responsive health care system and encompass the effects of socioeconomic and environmental factors on new-borns and mothers. Ethiopia is working to achieve the Sustainable Development Goal target for the reduction of 12 or less per 1000 birth by 2030 and 21 per 1000 livebirths by 2025 as part of the second Ethiopian Health Sector Transformation Plan.

**Objective:**

This study aimed to compare the performance of classical time-series models with that of deep learning models and to forecast the neonatal mortality rate in Ethiopia to verify whether Ethiopia will achieve national and international targets.

**Methods:**

Data were extracted from the official World Bank database. Classical time-series models, such as autoregressive integrated moving average (ARIMA) and double exponential smoothing, and neural network-based models, such as multilayer perceptron, convolutional neural network, and long short-term memory, have been applied to forecast neonatal mortality rates from 2021 to 2030 in Ethiopia. During model building, the first 21 years of data (from 1990 to 2010) were used for training, and the remaining 10 years of data were used to test model performance. Model performance was evaluated using *R*², mean absolute percentage error (MAPE), and root mean squared error (RMSE). Finally, the best model was used to forecast the neonatal mortality rate over the next 10 years from 2021 to 2030, with a 95% prediction interval (PI).

**Results:**

The results showed that the double exponential smoothing model was the best, with a maximum *R*^2^ of 99.94% and minimum MAPE and RMSE of 0.002 and 0.0748, respectively. The worst performing among the 5 models was the CNN, with an *R*^2^ of 93.71% and a maximum RMSE of 0.79. Neonatal mortality in Ethiopia is forecasted to be 23.20 (PI 22.20-24.40) per 1000 live births in 2025 and 19.80 (PI 17.10-22.80) per 1000 live births in 2030.

**Conclusions:**

This study revealed that national and international targets for neonatal mortality cannot be realized if the current trend continues. This highlights the need for urgent interventions to strengthen the health system to fasten the decline rate of neonatal mortality and collaborative effort with concerned stakeholders for improved and responsive neonatal and child health services in order to achieve these targets.

## Introduction

The neonatal mortality rate refers to the number of deaths during the first 28 days of life per 1000 live births, and it remains a significant global public health problem globally [1]. Neonatal diseases and their outcomes are important indicators of responsive health care systems and encompass the effects of socioeconomic and environmental factors on newborns and mothers. Although prevention and treatment are simple, relatively inexpensive, and efficient, the contribution of neonatal mortality to overall mortality and morbidity is overwhelming and unequaled by any other condition, as no age group has received less attention than neonates. Notwithstanding efforts to achieve the United Nations’ Millennium Development Goals (MDGs) and Sustainable Development Goals (SDGs) over the past 2 decades, children are still most likely to die during the neonatal period [2].

In 2019, 70% of deaths among children and youth aged 25 years and younger occurred in those aged 5 years and younger, with neonatal deaths accounting for a larger share of under-5 mortality. The contribution of neonatal mortality to all under-5 deaths increased from 40% in 1990 to 47% in 2019 [3]. Globally, 53 countries will not achieve the SDG target for under-5 mortality by 2030 if the trend evident from 2010 to 2019 continues. To meet these targets, resources and policies must be mobilized to accelerate progress rather than sustain the current declining trend.

Many low- and middle-income countries have reduced the number of children who die before the age of 5 years as a result of interventions undertaken since 1990 to meet MDG 4, which aimed at a two-thirds reduction in the mortality risks of children ages 5 years and younger by 2015. Despite the success achieved by MDGs in low- and middle-income countries, neonatal mortality is still unacceptably high, with the largest burden evident in sub-Saharan African countries [4]. According to a United Nations International Children's Emergency Fund (UNICEF) report, sub-Saharan African neonates were at tenfold greater risk of death than neonates born in developed countries. The report also showed that most neonatal deaths occurred in East Africa, more than 50% of them in Ethiopia [5].

In Ethiopia, a lower decline of 41% was observed in neonatal mortality compared to the 60% reduction in under-5 mortality and 50% reduction in infant mortality between 2000 and 2016 [6]. According to the SDG index, Ethiopia ranks 136 out of 166 countries, with an overall performance score of 55.2, which is above the regional average score of 53.1 [7]. This makes achievement of the SDG target to reduce the neonatal mortality rate to 12 per 1000 births by 2030, and the second Ethiopian Health Sector Transformation Plan (HSTP-II) target to reduce the neonatal mortality rate to 21 per 1000 live births by 2025 [8], questionable without special effort being made.

In this study, deep-learning models were applied in addition to classical time-series approaches to forecasting. Deep-learning models involve features such as the ability to handle temporal dependencies, distribution-free learning, and flexibility when modeling nonlinear features, which make them suitable for time-series data modeling [9]. This study aimed to compare the performance of commonly known classical time-series models with deep-learning models when forecasting the annual neonatal mortality rate in Ethiopia to verify whether Ethiopia achieves national and international targets. To the best of our knowledge, this paper presents a pioneering exploration of neonatal mortality forecasting using classical time-series and deep-learning techniques. Using historical data to predict future neonatal mortality is important for understanding trends and estimating the burden of neonatal mortality. The findings will help track Ethiopia’s progress toward achieving the SDG by 2030 and the national HSTP-II target by 2025.

## Methods

### Data Source

Neonatal mortality data used in this study were obtained from the World Bank’s Health Nutrition and Population Statistics database [10]. These data are compiled by the United Nations Inter-agency Group for Child Mortality Estimation (UN IGME), which includes the UNICEF, the World Health Organization, the World Bank, and the United Nations Department of Economic and Social Affairs (Population Division). The UN IGME uses a broad strategy to arrive at annual estimates of child mortality by compiling and assessing the quality of all available nationally representative data relevant to the estimation of child mortality, including data from vital registration systems, population censuses, household surveys, and sample registration systems [3]. Similarly, the neonatal mortality estimates are derived using standardized statistical models that synthesize data from a variety of sources, including national censuses, household surveys (such as Demographic and Health Surveys and Multiple Indicator Cluster Surveys), vital registration systems, and other country-reported data. These estimates are produced following rigorous validation methods to ensure comparability across countries and over time. For this study, we extracted Ethiopia’s national-level annual neonatal mortality rates for the period 1990 to 2020. These figures represent model-based estimates of the overall neonatal mortality rate in the country, expressed as the number of deaths within the first 28 days of life per 1000 live births per year. The data are publicly available through the UN IGME platform at [11].

### Data Processing and Analysis

Autoregressive integrated moving average (ARIMA) and double exponential smoothing from classical time-series models and multilayer perceptron (MLP), deep-learning algorithms such as convolutional neural networks (CNN), and long short-term memory (LSTM) were selected to forecast the neonatal mortality rate from 2021 to 2030 in Ethiopia.

### Classical Models

Classical time-series forecasting models, such as ARIMA and exponential smoothing, were applied using the well-known Python (Python Software Foundation) package, *statsmodels*.

**Figure 1.**
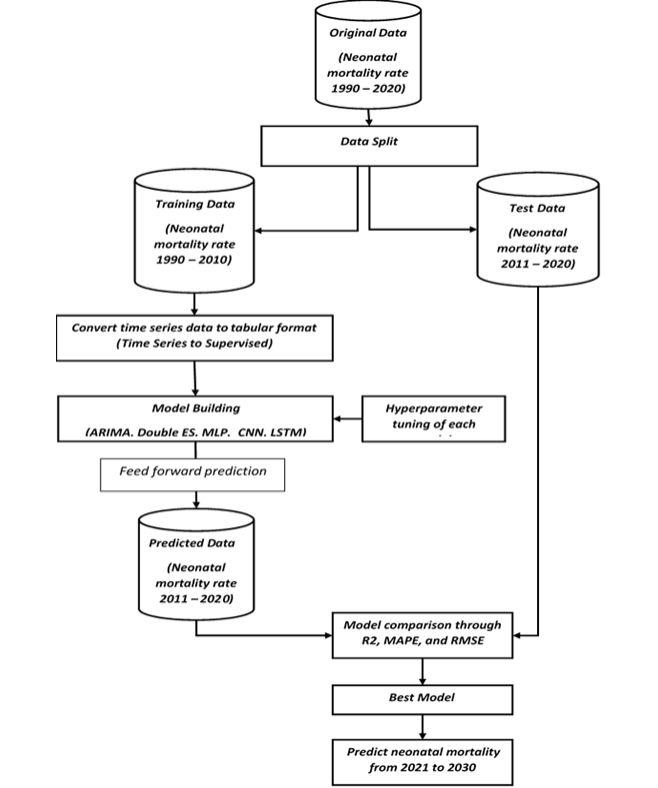
Workflow of data preparation and analysis plan to forecast neonatal mortality in Ethiopia. ARIMA: autoregressive integrated moving average; ES: Exponential smoothing; MLP: multilayer perceptron; CNN: convolutional neural network; LSTM: long short-term memory; MAPE: mean absolute percentage error; RMSE: root mean squared error.

ARIMA is a class of statistical models for analyzing and forecasting time-series data that incorporates the concept of integration and is a generalization of the straightforward autoregressive moving average. The autoregression (AR) part of ARIMA shows that the time series is regressed on its own past observations, the moving average part indicates that the forecast error is a linear combination of past respective errors, and the integrated (I) part shows that the data values have been replaced with different values of d to remove trends in the dataset and obtain stationary data, which is the assumption of the ARIMA model [12]. ARIMA forecasting is capable of dealing with nonstationary time-series data because of its “integrate” component that involves differencing the time series to convert a nonstationary time series into a stationary time series, which means that statistical properties such as mean, variance, autocorrelation, etc, are all constant over time. The general form of the ARIMA model is denoted as ARIMA (p, d, q) to represent the AR, I, and moving average components, respectively [13]. The difficulty of specifying p, d, and q (which is a significant limitation) can be overcome by experimenting with various combinations and assessing the model’s performance [12]. Hence, instead of using subjective diagnostic visualizations, such as the autocorrelation function and partial autocorrelation function, to determine these parameters (p, d, q), grid searching of specified values was performed, and the best combination that minimized the forecasting error was used to determine the best ARIMA model. ARIMA, which is a time series with a repeating cycle, has the drawback of not supporting the seasonal data. ARIMA expects data that are not seasonal or have had the seasonal component eliminated, such as data that have been seasonally adjusted using techniques such as seasonal differencing [14]. Fitting seasonal ARIMA (SARIMA), a version of the ARIMA model that incorporates additional seasonal components, is an alternative approach. However, Figure 1 shows that the data shows no repeating patterns.

The mathematical form of an ARIMA (p, d, q) model can be written as follows [15]:


$$y_{t}^{\prime }=\;c\;+\;\phi_{1}y_{t-1}^{\prime }+\;\phi_{2}y_{t-2}^{\prime }+\;\cdots +\;\phi_{p}y_{t-p}^{\prime }+\;\theta_{1}\epsilon_{t-1}+\;\theta_{2}\epsilon_{t-2}+\;\cdots +\;\theta_{q}\epsilon_{t-q}+\;\epsilon_{t}$$

Where:

$y_{t}^{`}$ is the differenced series after applying the integration part *d* times (ie, $y_{t}^{`}=y_{t}-y_{t-1}$, if *d*=1),

c is a constant,

$\phi_{1},\phi_{2},\ldots ,\phi_{p}$ are the coefficients for the autoregressive terms,

$\theta_{1},\theta_{2},\ldots ,\theta_{q}$ are the coefficients for the moving average terms,

$\epsilon_{t}$ is the error term (white noise) at time t,

$\epsilon_{t-1},\;\epsilon_{t-2},\;\ldots$ are past forecast errors.

Another classical time-series model used in this study is exponential smoothing. Time-series methods, such as the ARIMA family of methods, develop a model in which the prediction is a weighted linear sum of recent past observations. Exponential smoothing forecasting methods are similar in that a prediction is a weighted sum of past observations, but the model explicitly uses an exponentially decreasing weight for past observations; the more recent the observation, the higher is the associated weight [14]. There are 3 main types of exponential smoothing time-series forecasting methods: simple exponential smoothing, a simple method that assumes no systematic structure (ie, without a trend or seasonality); double exponential smoothing, an extension that explicitly handles trends; and triple exponential smoothing, which adds support to seasonality. Because our dataset had only a trend component, a double exponential smoothing model was fitted.

Double exponential smoothing requires parameters called alpha (α), also called the smoothing factor or smoothing coefficient, which controls the rate at which the influence of observations at previous time steps decays exponentially. α is often set between 0 and 1. A value close to 1 indicates fast learning (ie, only the most recent values influence the forecasts), whereas a value close to 0 indicates slow learning (ie, past observations have a large influence on forecasts). In addition to the α parameter, an additional smoothing factor, called beta (b or β), is added to control the decay of the influence of the change in trend. The method supports trends that change in different ways, additive and multiplicative, depending on whether the trend is linear or exponential, respectively. The damping coefficient Phi (p or φ) is another hyperparameter used to control the damping rate. Dampening means reducing the size of the trend over future time steps to a straight line (no trend), and it can be additive or multiplicative for a linear or exponential dampening effect. In general, double exponential smoothing has five hyperparameters: (1) alpha (α): smoothing factor for the level, (2) beta (β): smoothing factor for the trend, (3) trend type: additive or multiplicative, (4) dampen type: additive or multiplicative, and (5) phi (φ): damping coefficient.

The mathematical form of double exponential smoothing model can be written as follows [15]:


$${\hat{y}}_{t+h\vee t}=l_{t}+\left(\phi +\phi^{2}+\ldots +\;\phi^{h}\right)b_{t}$$

$$l_{t}=\;\alpha y_{t}+\left(1-\alpha \right)\left(l_{t-1}+\;\phi b_{t-1}\right)$$

$$b_{t}\;=\;\beta \left(l_{t}-\;l_{t-1}\right)+\left(1-\beta \right)\phi b_{t-1}$$

Where: *ℓt* denotes an estimate of the level of the series at time *t*,

*bt* denotes an estimate of the trend (slope) of the series at time

*t*, *h* denotes how many periods into the future we’re forecasting,

*α* is the smoothing parameter for the level, 0≤*α*≤1,

*β* is the smoothing parameter for the trend, 0≤*β*≤1, and

*ϕ* is the damping parameter 0<*ϕ*<1

Among them, the optimal values of alpha, beta, and phi can be automatically tuned by the model, and the optimal values of the trend- and dampen-type dampens are optimized by grid search hyperparameter tuning, as they could not be optimized automatically by the model.

### Deep Learning Algorithms for Time-Series Forecasting

Owing to their familiarity and versatility, linear techniques such as ARIMA have historically dominated time-series forecasting. However, these traditional approaches have certain drawbacks, such as the exclusion of complex joint distributions by assuming a linear relationship. However, machine-learning techniques can be useful for solving complex time-series forecasting issues that involve multiple input variables, sophisticated nonlinear relationships, and missing data.

In this study, the most basic type of feed-forward perceptron (MLP) and advanced deep learning algorithms, such as LSTM and CNN, were applied to forecast neonatal mortality in Ethiopia. All neural networks were built using the Keras deep-learning framework in Python.

### Data Preparation

The time-series dataset was split into 2 subsets, training and test datasets, whereby 68% of the dataset was used for training and the remaining 32% was used to test the accuracy of the models. Unlike the standard train-test split technique, which randomly splits the data, the temporality of the dataset should be maintained in time-series data partitioning. Hence, the first 21 years of data (from 1990 to 2010) were used for training, and the remaining 10 years of data (from 2011 to 2020) were used to test model performance.

As this study used univariate time-series data, the sequential nature of the data required preparation before it could be used to train a supervised learning model such as neural networks. Hence, the training data were prepared in tabular form and models were then developed. A supervised learning algorithm requires data to be provided as a collection of samples, where each sample has an input component (*X*) and output component (*y*). The number of time lags used as the independent variable (*X*) was determined by trying the number of time lags (n_input) and the time lag that minimized the forecasting error. The results of the hyperparameter tuning revealed that the best-performing model was obtained when the previous 4 observations (n_input=4) were used as independent variables to predict the fifth observation as the dependent variable. This means that the data from 1990 to 1993 was used as an input variable to predict the 1994 neonatal mortality rate, and the 1991 to 1994 rate was used to predict the neonatal mortality rate of 1995, and so on.

### Forecasting and Model Performance Assessment

Time-series forecasting models can be evaluated on a test set using walk-forward validation, an approach in which the model makes a forecast for each observation in the test set, one at a time. After each forecast was made for a time step in the test dataset, true observations for the forecast were added to the test dataset and made available to the model. Nevertheless, the true observation for the time step could then be used as part of the input to make a prediction for the next time step. Using this forecasting approach, 10 years’ worth of data (from 2011 to 2020) was predicted, the predictions were compared to the true values in the test set, and an error measure was calculated.

To evaluate the predictive performance of the forecasting method, commonly applicable measures, such as *R*^2^, root mean squared error (RMSE), and mean absolute percentage error (MAPE), were used. Accordingly, the mode that maximizes *R*^2^ or minimizes RMSE or MAPE is selected as the best model. Finally, the best model was used to forecast the neonatal mortality rate for the next 10 years, from 2021 to 2030, with a 95% prediction interval (PI). Figure 1 shows the overall workflow of data preparation and analysis.

### Ethical Considerations

This study used fully anonymized, aggregate-level data from the publicly accessible World Bank Health, Nutrition, and Population Statistics database. While there are no publicly available national or institutional policies in Ethiopia that explicitly address the ethics review requirements for such publicly available secondary data use, the nature of the data and the research process met the exemption criteria for ethics review under internationally recognized standards. Per the Government of Canada Panel on Research Ethics, as this work involved analysis of preexisting, publicly disseminated data, it did not involve any interaction with human participants and presented zero reidentification risk due to the aggregated nature of the data, this study did not require research ethics board review or approval per the Tri-Council Policy Statement: Ethical Conduct for Research Involving Humans (TCPS 2‐2022, Articles 2.2 and 2.4) [16]. Therefore, the study was conducted in compliance with recognized ethical standards for the use of public secondary data.

## Results

**Figure 2.**
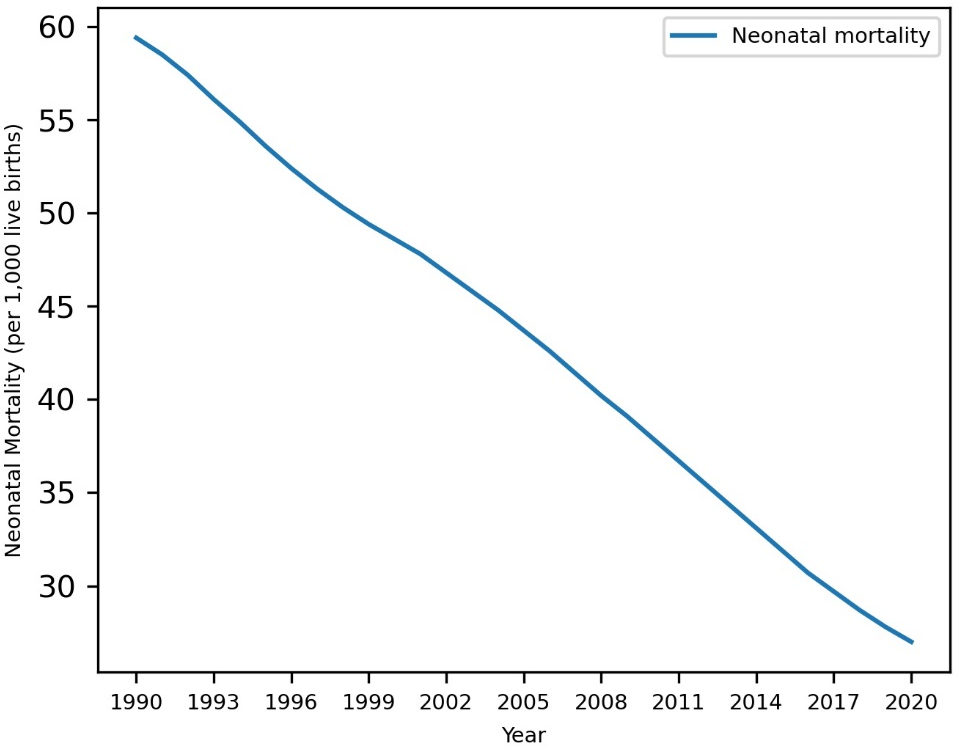
Neonatal mortality in Ethiopia (1990‐2020).

Neonatal mortality has declined significantly in Ethiopia, from 59.4 per 1000 live births in 1990 to 27 per 1000 live births in 2020. Figure 2 shows that neonatal mortality has shown a decreasing trend over time. This trend was eliminated by first-order differencing as the best order in the hyperparameter tuning result; hence, neonatal mortality after differencing was stationary and ready for forecasting.

### Hyperparameter Tuning

For each model applied in the analysis, grid search hyperparameter tuning was used to develop the best model with possible optimal values. Table 1 shows the hyperparameters tried and the optimal values that minimized the forecasting errors.

**Table 1.** Results of model hyperparameter tuning.

Model	Model hyperparameters^a^	Hyperparameter space^b^	Total combination	Best combination
ARIMA^c^	p, d, q	q=[0,1,2,3] d=[0,1,2,3] p=[0,1,2,3,4]	80	p=2 d=1 q=2
Double ES^d^	trend damped	trend=['add', 'mul'] damped=[True, False]	4	trend= 'add’ damped=True
MLP^e^	n_input n_nodes n_batch n_diff	n_input = [2-5] n_nodes = [100, 150] n_batch = [1, 150] n_diff = [0,1, 2,4]	64	n_input=4 n_nodes=100 n_batch=1 n_diff=1
LSTM^f^	n_input n_nodes n_batch n_diff	n_input=[2-5] n_nodes=[100, 150] n_batch=[1, 150] n_diff=[0,1, 2,4]	64	n_input=4 n_nodes=100 n_batch=1 n_diff=1
CNN^g^	n_input n_diff	n_input=[2-5] n_diff=[0,1, 2,4]	16	n_input=4 n_diff=1

a Hyperparameters are model-specific settings tuned to optimize performance.

b Values in brackets represent the range or options tested using grid search.

c ARIMA: autoregressive integrated moving average.

d ES: exponential smoothing.

e MLP: multilayer perceptron.

f LSTM: long short-term memory.

g CNN: convolutional neural network.

The hyperparameters of all the models were tuned in a grid search manner, and the best combination of hyperparameters was used to develop the final model. Accordingly, the lag order (p), degree of differencing (d), and order of the moving average (q) are the hyperparameters of ARIMA. Similarly, the trend and damped hyperparameters of double exponential smoothing were tuned.

The number of previous inputs to be used as inputs for the model (n input), the number of nodes to be used in the hidden layer (n_nodes), and the number of samples to be included in each mini-batch (n_ batch) were the tuned hyperparameters for neural networks (ie, MLP, LSTM, and CNN). Furthermore, the difference order (n_diff) was tuned to obtain the optimal value for removing the trend from the data. The n_diff optimal value for all neural networks is 1, suggesting that the value of a previous observation should be subtracted from the current observation before fitting the model and that the predictions of the model need to be reversed to obtain the normal forecasted data. Table 1 presents the full range of hyperparameters tested for each model (the hyperparameter space), the total number of combinations evaluated, and the best-performing configuration based on model evaluation criteria (eg, lowest RMSE or MAE). The hyperparameters listed in the table correspond to parameters in the Python libraries used in this study: *statsmodels* for classical models (ARIMA and Exponential Smoothing) and *Keras* for deep learning models (MLP, LSTM, and CNN).

### Model Comparison

After developing the final models based on the optimal hyperparameters of the training data, the performance of the fitted models on the test data was assessed, as presented in Table 2. The results showed that the double exponential smoothing model was the best, with a maximum *R*^2^ of 99.94% and minimum MAPE and RMSE of 0.0015 and 0.0748, respectively. The worst-performing among the five models was the CNN, with an *R*^2^ of 93.71% and a maximum RMSE of 0.7903.

**Figure 3.**
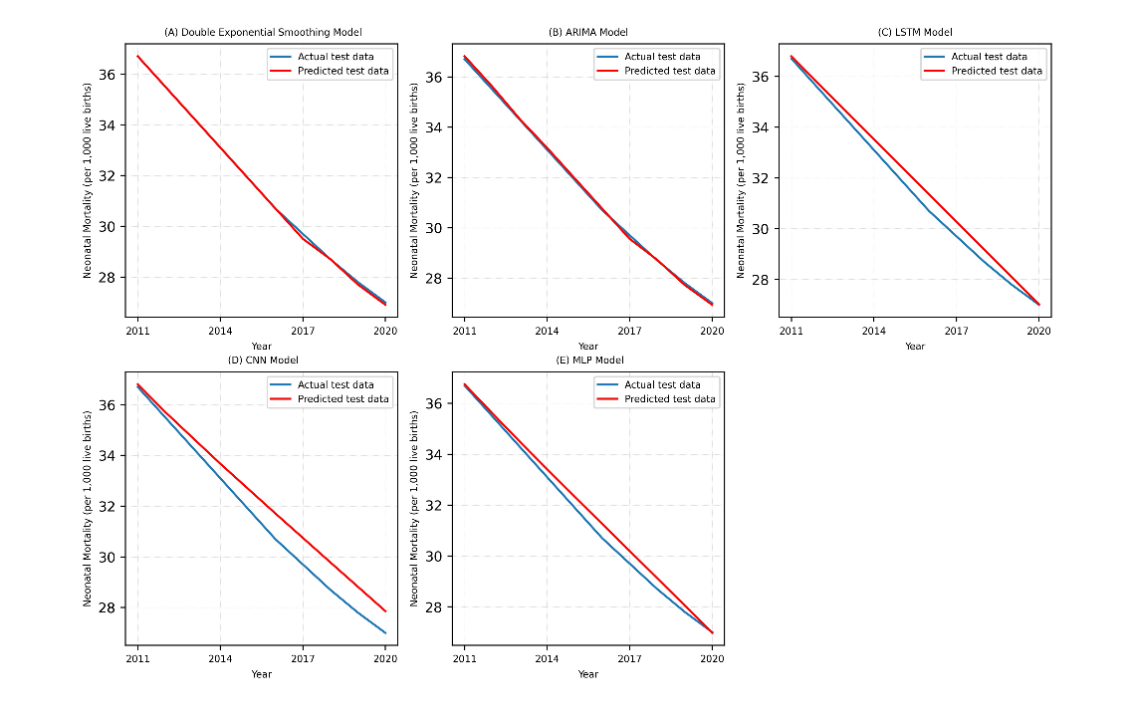
Performance comparison of classical and deep learning models on the test data. ARIMA: autoregressive integrated moving average; LSTM: long short-term memory; CNN: convolutional neural network; MLP: multilayer perceptron.

This performance was also clearly observed graphically, with an overlap of the predicted and actual data in double exponential smoothing and a large gap in the CNN model (see Figure 3).

**Table 2.** Model performance comparison results.

Model	*R* ^2^	MAPE^a^	RMSE^b^
Double exponential smoothing	0.9994	0.0015	0.0748
ARIMA^c^	0.9992	0.0025	0.0864
MLP^d^	0.9884	0.0094	0.3396
LSTM^e^	0.9830	0.0116	0.4110
CNN^f^	0.9371	0.0237	0.7903

a MAPE: mean absolute percentage error.

b RMSE: root mean squared error.

c ARIMA: autoregressive integrated moving average.

d MLP: multilayer perceptron.

e LSTM: long short-term memory.

f CNN: convolutional neural network.

### Final Prediction

**Figure 4.**
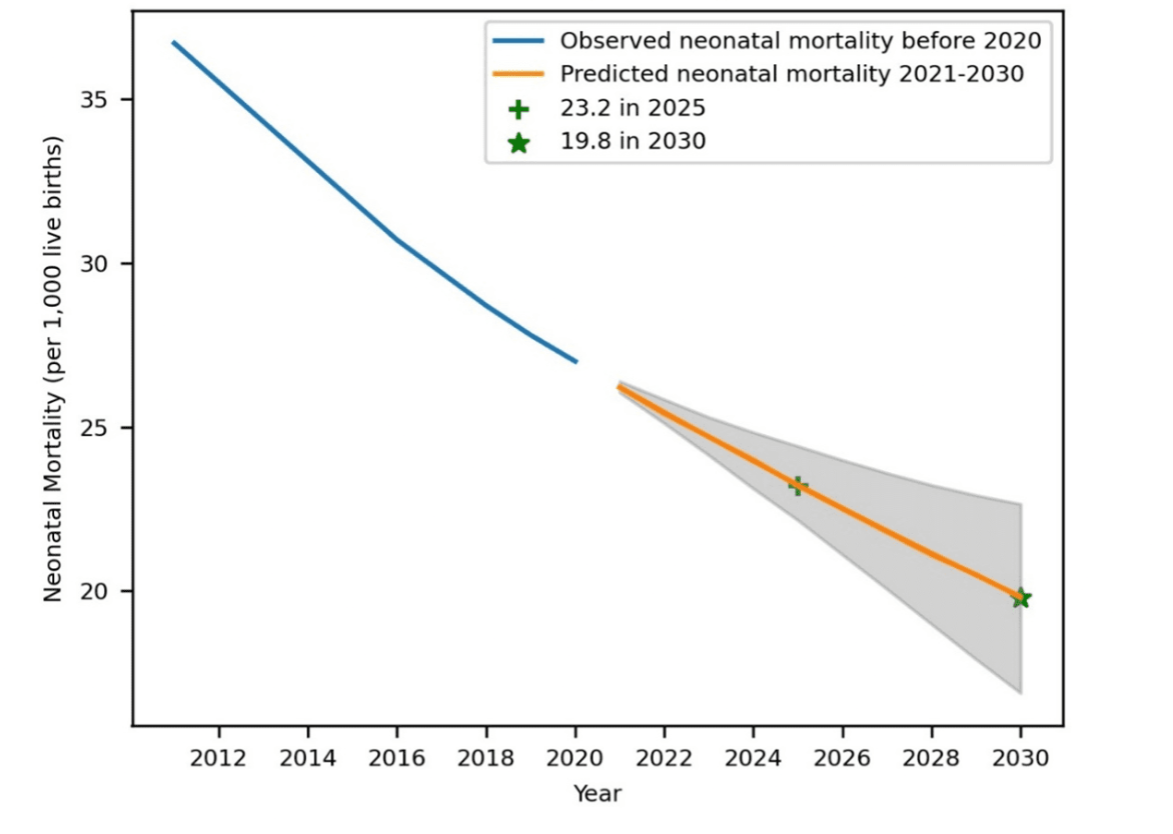
Prediction of neonatal mortality in Ethiopia from 2021 to 2030.

Neonatal mortality was predicted from 2021 to 2030 using a double exponential smoothing model with a 95% PI (see Table 3 and Figure 4). Accordingly, neonatal mortality in Ethiopia was forecasted to be 23.2 (PI 22.2-24.4) per 1000 live births in 2025 and 19.8 (PI 17.1-22.8) per 1000 live births in 2030. From this estimation, it is clear that Ethiopia is slightly behind what is necessary to achieve the HSTP-II neonatal mortality rate target of 21 per 1000 live births by 2025 and is also highly unlikely to meet the SDG target of 12 or fewer neonatal deaths per 1000 live births by 2030.

**Table 3.** Neonatal mortality forecasting in Ethiopia (2021 to 2030).

Year	Predicted neonatal mortality, deaths per 1000 live births (PI^a^)
2021	26.2 (26.1-26.4)
2022	25.4 (25.1-25.8)
2023	24.7 (24.2-25.2)
2024	24.0 (23.2-24.8)
2025	23.2 (22.2-24.4)
2026	22.5 (21.2-23.9)
2027	21.8 (20.2-23.6)
2028	21.1 (19.1-23.4)
2029	20.5 (18.1-23.1)
2030	19.8 (17.1-22.8)

a PI: prediction interval.

## Discussion

### Principal Findings

In this study, the performance of deep-learning and classical time-series models in predicting neonatal mortality rates in Ethiopia was compared. The methods included ARIMA, exponential smoothing, MLP, CNN, and LSTM. The comparison was based on evaluation metrics such as *R*^2^, MAPE, and RMSE. Based on these results, exponential smoothing performed best, with the lowest MAPE and RMSE values of 0.0015 and 0.0748, respectively. ARIMA was also the best-performing model, followed by exponential smoothing, for predicting neonatal mortality. However, the CNN performed the worst among the 5 models. This result is compatible with previous studies that reported that classical methods are more accurate than neural network models [17-19]. This result is also in line with a study that reported that LSTMs are easily outperformed by simpler methods and may not be suitable for simple autoregressive-type univariate time-series forecasting tasks [20]. In a study aimed at clearly demonstrating the capability of a suite of different machine-learning methods compared to classical time-series forecasting methods evaluated across a collection of 1045 monthly time series, simple classical methods such as exponential smoothing and ARIMA models outperformed the machine-learning methods, including 2 modern neural network algorithms, such as recurrent neural networks and LSTM [21]. This may be owing to the appropriateness of classical methods in the case of univariate small datasets, where deep-learning algorithms are not yet at their best [22].

Another aim of this study was to forecast neonatal mortality to verify whether Ethiopia has achieved national and international targets. The results revealed that neonatal mortality in Ethiopia will be 23.2 (PI 22.2-24.4) per 1000 live births in 2025 and 19.8 (PI 17.1-22.8) per 1000 live births in 2030. These results reveal Ethiopia’s inability to achieve the SDG of reducing neonatal mortality, possibly because the COVID-19 pandemic and the armed conflict that occurred in 2019 jeopardized the health care system and made it difficult for women to receive prenatal and postnatal care due to lockdowns and restrictions, which in turn created vulnerable populations, exposing expectant mothers and newborns to danger. Furthermore, ongoing conflict in some areas of Ethiopia means that some health care facilities are nonoperational or services are interrupted, which consequently makes it difficult to achieve the SDG. The risk of neonatal infections and malnutrition may further increase in conflict zones due to inadequate sanitization, food insecurity, and access to clean water. The ability to mitigate these issues and create stable environments, which is needed to make significant advancements in lowering neonatal mortality in Ethiopia, depends on the peaceful resolution of conflicts and humanitarian aid.

### Limitations

One of the primary limitations of this study is the relatively small size of the dataset, which includes annual neonatal mortality rates from 1990 to 2020. While this limited data span presents challenges, such as an increased risk of model overfitting and higher prediction uncertainty, we have taken extensive measures to address these issues. Specifically, we have applied advanced optimization techniques, including rigorous regularization, hyperparameter tuning, and cross-validation, to ensure that our models are robust and effectively fit the data. These efforts help to mitigate the impact of the small dataset on the accuracy and reliability of our forecasts for the period from 2021 to 2030. Although the dataset size constrains the breadth of variability and external factors captured, our careful optimization and validation processes enhance the credibility of the results. Future research could build on this foundation by incorporating larger and more diverse datasets, which would further strengthen the robustness of predictive models and provide even more accurate forecasts.

### Conclusion

This study found that classical models outperformed advanced neural network models in forecasting neonatal mortality in Ethiopia. This implies the need to use classical time-series methods in time-series forecasting rather than relying on advanced deep-learning models. This study also revealed that the national (HSTP-II) and international (SDG) targets for neonatal mortality cannot be realized if the current trend continues. This highlights that urgent interventions are necessary to strengthen the health care system and accelerate the decline in the neonatal mortality rate. To achieve these targets, collaborative efforts with concerned stakeholders are also needed to improve the health care system and provide responsive neonatal and child health services.

## Abbreviations

ACF: autocorrelation function
ARIMA: autoregressive integrated moving average
CNN: convolutional neural network
HSTP-II: second Ethiopian Health Sector Transformation Plan
LSTM: long short-term memory
MA: moving average
MAPE: mean absolute percentage error
MDG: Millennium Development Goals
MLP: multilayer perceptron
PACF: partial autocorrelation function
PI: prediction interval
RMSE: root mean squared error
SDG: Sustainable Development Goal
UN IGME: United Nations Inter-agency Group for Child Mortality Estimation
UNICEF: United Nations International Children's Emergency Fund
